# Different cholinergic cell groups in the basal forebrain regulate social interaction and social recognition memory

**DOI:** 10.1038/s41598-021-93045-7

**Published:** 2021-06-30

**Authors:** Kana Okada, Kayo Nishizawa, Tomoko Kobayashi, Shogo Sakata, Kouichi Hashimoto, Kazuto Kobayashi

**Affiliations:** 1 Department of Neurophysiology, Graduate School of Biomedical and Health Sciences, Hiroshima University, Hiroshima, 734-8551 Japan; 2 Career Assistant Project, Hiroshima University, Higashi-Hiroshima, 739-8521 Japan; 3 Department of Molecular Genetics, Institute of Biomedical Sciences, Fukushima Medical University School of Medicine, Fukushima, 960-1295 Japan; 4 Department of Behavioural Sciences, Graduate School of Humanities and Social Sciences, Hiroshima University, Higashi-Hiroshima, 739-8521 Japan

**Keywords:** Molecular biology, Neuroscience, Neurology

## Abstract

Social behaviour is a complex construct that is reported to include several components of social approach, interaction and recognition memory. Alzheimer’s disease (AD) is mainly characterized by progressive dementia and is accompanied by cognitive impairments, including a decline in social ability. The cholinergic system is a potential constituent for the neural mechanisms underlying social behaviour, and impaired social ability in AD may have a cholinergic basis. However, the involvement of cholinergic function in social behaviour has not yet been fully understood. Here, we performed a selective elimination of cholinergic cell groups in the basal forebrain in mice to examine the role of cholinergic function in social interaction and social recognition memory by using the three-chamber test. Elimination of cholinergic neurons in the medial septum (MS) and vertical diagonal band of Broca (vDB) caused impairment in social interaction, whereas ablating cholinergic neurons in the nucleus basalis magnocellularis (NBM) impaired social recognition memory. These impairments were restored by treatment with cholinesterase inhibitors, leading to cholinergic system activation. Our findings indicate distinct roles of MS/vDB and NBM cholinergic neurons in social interaction and social recognition memory, suggesting that cholinergic dysfunction may explain social ability deficits associated with AD symptoms.

## Introduction

Social behaviour is a complex construct and may include several components for social approach, interaction, and recognition memory^1–3^. Previous studies have reported that social behaviour requires the function of multiple brain regions, such as the medial prefrontal cortex^4–7^. The cholinergic system is a potential constituent for the neural mechanisms underlying social behaviour^8–10^. Indeed, systemic administration of the muscarinic acetylcholine receptor antagonist scopolamine disrupts social interaction or social recognition in rodents^11–13^, and deficits in these behaviours were restored by treatment with the acetylcholinesterase inhibitor galantamine or the nicotinic acetylcholine receptor agonist AR-R 17779^12,13^.

The most prominent characteristic of Alzheimer’s disease (AD) is mnemonic dysfunction, but along with the progress of the disease, behavioural and psychological symptoms of dementia (BPSD), including social illness, are commonly seen in patients^14,15^. Mnemonic dysfunction in AD is considered to be mostly caused by the substantial loss of acetylcholine-containing neurons in the basal forebrain^16–18^, and a number of experimental studies in animals support the finding that basal forebrain cholinergic neurons play a crucial role in mnemonic function^19,20^. In addition, previous studies have suggested that some BPSD, including impaired social ability, also appear to have a cholinergic basis^21,22^. However, the involvement of basal forebrain cholinergic system in social behaviour is not yet fully understood.

The basal forebrain cholinergic system is comprised of discrete cell groups that innervate a variety of brain regions; neurons in the medial septum (MS) and vertical diagonal band of Broca (vDB) provide cholinergic projections predominantly to the hippocampus and the medial prefrontal cortex, and neurons in the nucleus basalis magnocellularis (NBM) provide cholinergic innervations to the entire cortex and basolateral amygdala^23–26^. These anatomical findings that cholinergic projection areas overlap with the brain regions required for social behaviour indicate the possibility that basal forebrain cholinergic cell groups may be involved in the control of social function through modulation of the activity of the related brain regions.

To address the role of MS/vDB and NBM cholinergic neurons in the basal forebrain in social behaviour, we carried out immunotoxin (IT)-mediated cell targeting^27,28^ to selectively eliminate each cholinergic cell group, and then tested social behaviour by using the three-chamber test that was developed as the standardized assay for the assessment of social interaction and social recognition memory in mice^3^. We used transgenic (Tg) mice that express a chimeric gene containing human interleukin-2 receptor α-subunit (IL-2Rα) and a variant of yellow fluorescent protein (mVenus) under the control of the murine cholineacetyltransferase gene promoter (termed *ChAT-IL-2Rα/mVenus* Tg mice), and IT injection into the MS/vDB or NBM of *ChAT-IL-2Rα/mVenus* Tg mice removed selectively the respective cholinergic cell groups in Tg mice without damage in parvalbumin-positive neurons^20^. This elimination of cholinergic neurons also led to the decrease in synaptic terminals of these neurons projecting to the hippocampus and the cerebral cortices in the IT-injected Tg mice into the MS/vDB and NBM, respectively^20^. In the present study, elimination of MS/vDB cholinergic neurons damaged the preference for social stimuli over non-social stimuli, indicating reduction in social interaction, whereas it did not alter the detection of novel social stimuli, showing the intact social recognition memory. In contrast, NBM cholinergic elimination, although persisted normally social interaction, resulted in impaired social recognition memory. The decline in social interaction and social recognition memory in the injected mice was restored by cholinergic activation with cholinesterase inhibitors (ChEIs), such as donepezil (Done) and rivastigmine (Riva). Our results indicate that MS/vDB and NBM cholinergic neurons have important roles in different types of social behaviour, suggesting that deficits in basal forebrain cholinergic systems may explain impairment in social ability related to AD symptoms.

## Results

### Selective targeting of MS/vDB and NBM cholinergic cell groups

**Figure 1:**
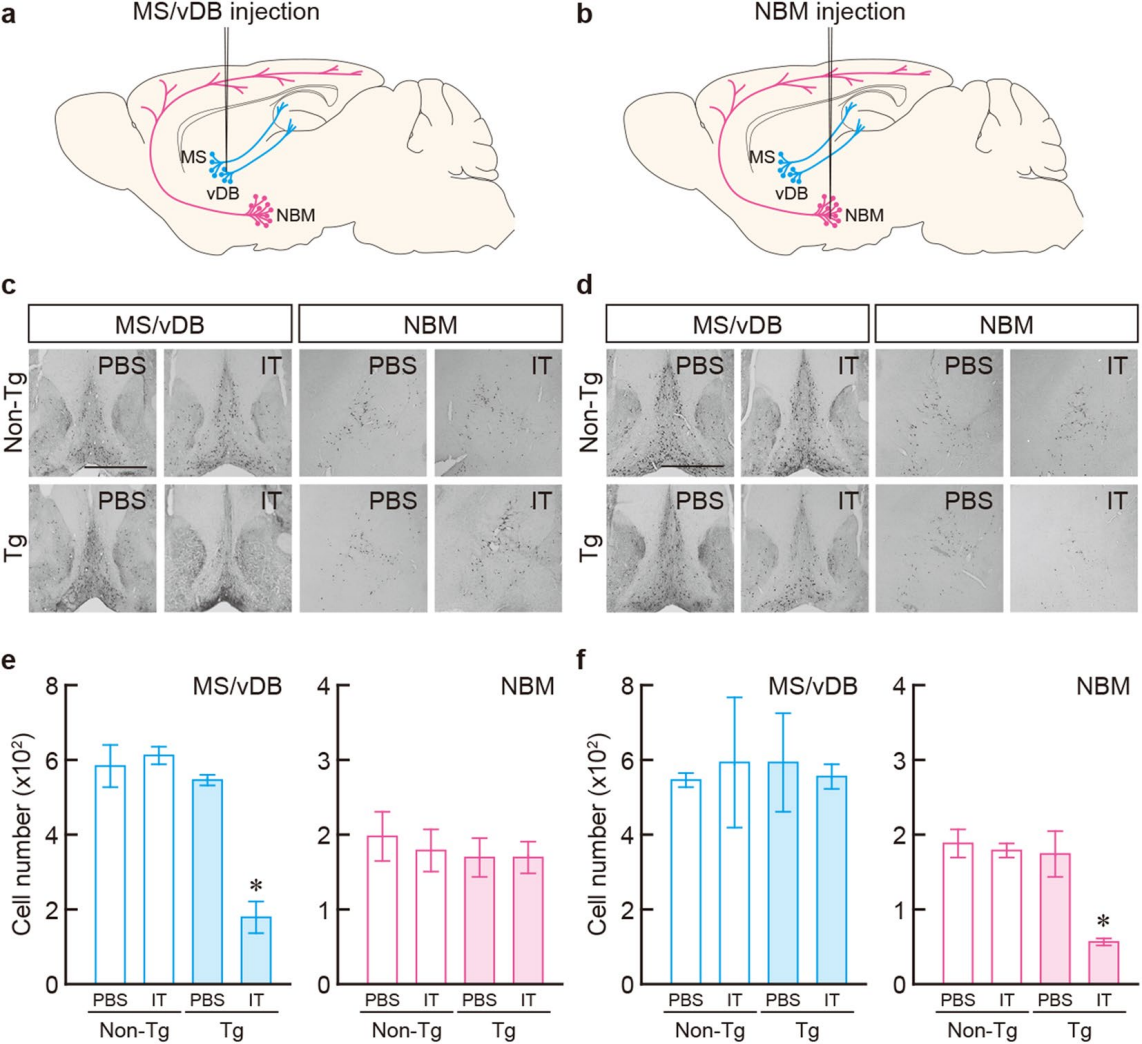
Selective targeting of basal forebrain cholinergic cell groups. (**a,b**) Schematic representation of the intracranial IT injection by using stereotaxic surgery into the MS/vDB (**a**) or NBM (**b**). Black needles indicate the glass pipets for the injection. (**c,d**) ChAT immunostaining with sections through the MS/vDB or NBM prepared from the Tg and non-Tg mice 7 days after IT/PBS injection into the MS/vDB (**c**) or NBM (**d**). Scale bars: 1 mm. (**e,f**) Cell counts of ChAT-positive neurons in the MS/vDB or NBM in the injected mice into the MS/vDB (**e**) or NBM (**f**). Data are presented as mean ± s.e.m. *n* = 3 for each group. *P < 0.05 vs each of other three groups (*Bonferroni* method).

We performed selective elimination of cholinergic neurons in the basal forebrain by using IT-mediated cell targeting^27,28^. Tg mice were generated that carry a chimeric gene encoding human interleukin-2 receptor α-subunit (IL-2Rα) fused to a variant of enhanced yellow fluorescent protein (mVenus) under the control of the gene promoter for choline acetyltransferase (ChAT), and the IL-2Rα/mVenus transgene was expressed in the majority of ChAT-positive neurons in both the MS/vDB and NBM regions of the *ChAT-IL-2Rα/mVenus* mice, as described in our previous study^20^. Anti-Tac(Fv)-PE38, which consists of single-chain variable regions of a monoclonal antibody for human IL-2Rα connected to a bacterial exotoxin catalytic fragment^29^, was used as a recombinant IT for intracranial injection into the basal forebrain regions (Fig. 1a, b). Tg and non-transgenic (non-Tg) mouse littermates (8–10 weeks old, n = 12 for each mouse group) were given intracranial injections of IT (20 μg/ml) or phosphate-buffered saline (PBS) into the MS/vDB (0.2 μl × 12 sites) or NBM (0.3 μl × 6 sites) (see Supplementary Fig. S1 for the injection sites). One week after the surgery, the brains were processed for immunohistochemistry. Sections through the basal forebrain were immunostained by using anti-ChAT antibody, and were viewed for cell counts. IT injection into the MS/vDB or NBM resulted in a loss of ChAT-positive neurons in the corresponding regions in the Tg mice only (Fig. 1c,d). One-way analysis of variance (ANOVA) for the mice that received injection into the MS/vDB indicated a significant difference in the number of cells in the MS/vDB among the four mouse groups (Fig. 1e; *F*_3,8_ = 26.567, *P* < 0.001), with a significant reduction in the IT-injected Tg mice compared with each of other three groups (*Bonferroni* method, *P* < 0.05). The percentage of the mean number of MS/vDB cells in the IT-injected Tg mice was 31% of that in the PBS-injected non-Tg mice. The reduction of cholinergic cell number in the MS/vDB was observed along with the anteroposterior axis (Supplementary Fig. S2). However, no significant difference in the cell number in the NBM was observed among the mouse groups (Fig. 1e). For the mice injected into the NBM, one-way ANOVA followed by post hoc multiple comparisons revealed that the NBM cell number was significantly lower in the IT-injected Tg mice compared with those in each of other three groups (Fig. 1d; *F*_3,8_ = 11.316, *P* = 0.003, *Bonferroni* method, *P* < 0.05), and the percentage of the mean number of NBM cells in the IT-injected Tg mice was 30% of that in the PBS-injected non-Tg mice. The reduced cell number in the NBM was observed along with the anteroposterior axis (Supplementary Fig. S2). There was no difference in the MS/vDB cell number among the mouse groups (Fig. 1f). These findings confirm the selective and efficient elimination of cholinergic neurons in the basal forebrain regions of the Tg mice after IT injection.

### Targeting MS/vDB and NBM neurons impairs different types of social behaviour

**Figure 2:**
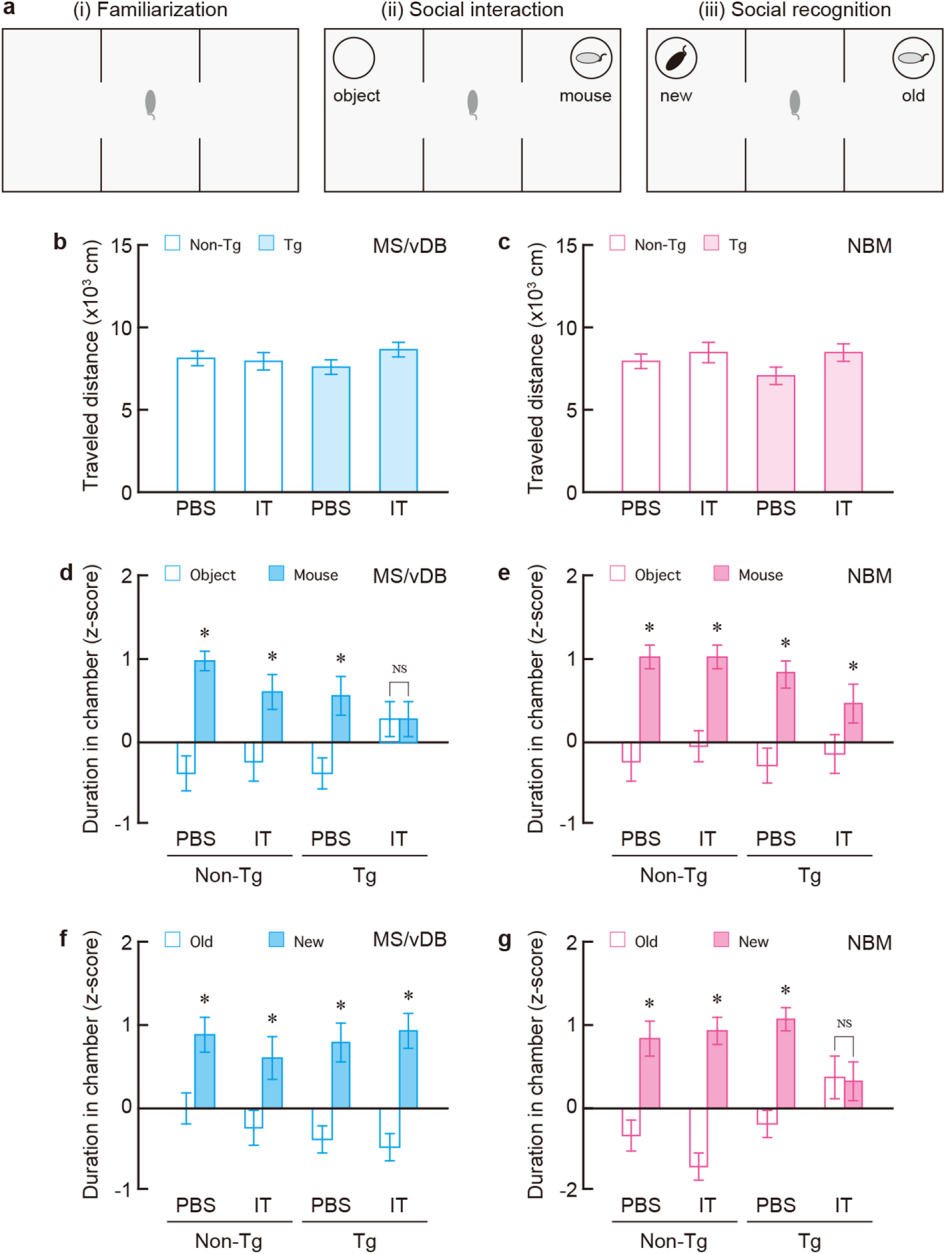
Elimination of the MS/vDB and NBM cholinergic neurons impaired social interaction and social recognition memory, respectively. Tg and non-Tg mice were injected with IT solution or PBS into the MS/vDB or NBM and then performed for the behavioural test. (**a**) Strategy for the three-chamber test. The large square represents the rectangle open field, which was divided into three chambers (a central chamber and two side chambers). The bold circled areas indicate the cages. The mice individually partook in the three successive 10-min sessions for (i) familiarization, (ii) social interaction, and (iii) social recognition memory, with 1-min intersession intervals within one day. (**b,c**) Mean travelled distance in the apparatus during the familiarization session in the injected mice into the MS/vDB (**b**) or NBM (**c**). Data are presented as mean ± s.e.m. *n* = 15 for each group. (**d,e**) Mean z-score of the duration in the chambers with the mouse and the object cages during the social interaction session in the injected mice into the MS/vDB (**d**) or NBM (**e**). Data are presented as mean ± s.e.m. *n* = 15 for each group. *P < 0.05 vs the object chamber (two-way ANOVA with the repeated measure). NS, not significant. (**f,g**) Mean z-score of the duration in the chambers with familiar mouse (old) and novel mouse (new) during the social recognition memory session in the injected mice into the MS/vDB (**f**) or NBM (**g**). Data are presented as mean ± s.e.m. *n* = 15 for each group. *P < 0.05 vs the old mouse chamber (two-way ANOVA with the repeated measure). NS, not significant.

Tg and non-Tg mice (8–10 weeks old, n = 60 for each mouse group) were administered PBS or IT injection into the MS/vDB or NBM, and were subjected to the three-chamber test that was developed as a standardized assay for the assessment of social interaction and social recognition memory^3^. The test was carried out using apparatus that consisted of three open-field adjacent chambers (a central chamber and two side chambers) separated by two clear plastic dividers, and connected by open doorways (Fig. 2a). The procedure consisted of three 10-min sessions for (i) familiarization, (ii) social interaction, and (iii) social recognition memory with 1-min intersession intervals. In the first session, familiarization (Fig. 2a–i), the mice were placed in the central chamber, allowed to habituate to the apparatus, and freely explored it. To monitor the locomotor activity of the mouse groups, the travelled distance in the apparatus was measured by using a video tracking system. The distance was similar among the four mouse groups that received the MS/vDB injection (Fig. 2b: *F*_3,56_ = 0.956, *P* = 0.420, one-way ANOVA) or NBM injection (Fig. 2c: *F*_3,56_ = 1.461, *P* = 0.235, one-way ANOVA), showing normal locomotor activity during the familiarization session in the mice with elimination of the MS/vDB or NBM cell group.

In the second session for social interaction (Fig. 2a-ii), a steel meshed cage with an unfamiliar C57BL/6 J male mouse (8–10 weeks old) was placed in one of two side chambers (mouse chamber), and another identical empty cage was placed as an object in the other side chamber (object chamber). The duration of how long an injected mouse would stay in each chamber was measured, and the value in the mouse chamber against the object chamber was compared, because animals normally prefer to explore social stimuli than objects. To alleviate the influence of baseline locomotor activity variability among individual animals on the analysis of social behaviours, the data were normalized as z-scores according to the formula: $z=\frac{X-\bar{X}}{S}$, where $X$ was the raw score of duration in each chamber, $\bar{X}$ is the mean among the durations in all chambers, and $S$ is the standard deviation. For the MS/vDB injection, the duration in the chamber in the three mouse groups except for the IT-injected Tg mice showed higher scores for the mouse chamber compared to those for the object chamber. The scores were not significantly different between the two chambers in the IT-injected Tg group only (Fig. 2d: group, *F*_3,56_ = 0.519, *P* = 0.671; chamber, *F*_1,56_ = 23.692, *P* < 0.001; interaction, *F*_3,56_ = 3.566, *P* = 0.020, two-way ANOVA; non-Tg/PBS, *F*_1,56_ = 20.331, *P* < 0.001; non-Tg/IT, *F*_1,56_ = 7.679, *P* = 0.008; Tg/PBS, *F*_1,56_ = 9.380, *P* = 0.003; Tg/IT, *F*_1,56_ < 0.001, *P* = 0.992, post hoc). For the NBM injection, the duration in the four mouse groups showed the larger scores for the mouse chamber compared to the object chamber (Fig. 2e: group, *F*_3,56_ = 2.037, *P* = 0.119; chamber, *F*_1,56_ = 44.702, *P* < 0.001; interaction, *F*_3,56_ = 1.017, *P* = 0.392, two-way ANOVA). These data suggest that elimination of the MS/vDB cholinergic cell group resulted in impairment of social interaction, whereas NBM cholinergic ablation did not influence the interaction in the three-chamber test.

In the third session, for social recognition memory (Fig. 2a-iii), the cage with the male mouse in the second session was kept in the same side chamber (old mouse chamber), in which the mouse becomes familiar one. Another identical cage with a new, unfamiliar C57BL/6 J male mouse (8–10 weeks old) was placed in the side chamber (new mouse chamber) as opposed to the empty cage that was used in the second session. The duration in the chamber of the injected mouse was monitored, and the preference for the new mouse chamber over the old mouse chamber was compared, since animals normally prefer to explore novel social stimuli than familiar social stimuli, depending on mnemonic function to differentiate between these two stimuli. As in the second session, the duration data were normalized as z-scores. For the MS/vDB injection, chamber durations in the four mouse groups exhibited higher scores for the new mouse chamber compared to the old mouse chamber (Fig. 2f: group, *F*_3,56_ = 1.068, *P* = 0.370; chamber, *F*_1,56_ = 39.066, *P* < 0.001; interaction, *F*_3,56_ = 0.528, *P* = 0.665, two-way ANOVA). For the NBM injection, the duration in the mouse groups other than the IT-injected Tg mouse group had greater scores for the new mouse chamber compared to the old mouse chamber, but there was no significant difference in the scores between the two chambers in the IT-injected Tg group (Fig. 2g: group, *F*_3,56_ = 1.676, *P* = 0.183; chamber, *F*_1,56_ = 43.599, *P* < 0.001; interaction, *F*_3,56_ = 5.902, *P* = 0.001, two-way ANOVA; non-Tg/PBS, *F*_1,56_ = 14.993, *P* < 0.001; non-Tg/IT, *F*_1,56_ = 28.925, *P* < 0.001; Tg/PBS, *F*_1,56_ = 17.355, *P* < 0.001; Tg/IT, *F*_1,56_ = 0.044, *P* = 0.835, post hoc). These results suggest that cholinergic elimination of the NBM cell group impairs social recognition memory, while sustaining recognition memory for MS/vDB cholinergic removal.

Our findings obtained from the behavioural analysis with the three-chamber test demonstrate the functional double dissociation of basal forebrain cholinergic cell groups in social behaviour, revealing distinct roles of cholinergic neurons in the MS/vDB and NBM in social interaction and social recognition memory, respectively.

### Impaired social behaviour is recovered by pharmacological cholinergic activation

We examined whether impairment in social interaction or social recognition memory of mice lacking basal forebrain cholinergic cell groups could be recovered by pharmacological cholinergic activation with ChEIs that inhibit the degradation of acetylcholine, including Done and Riva. These drugs are known to be anti-dementia agents for AD^30–33^, and proved to be effective on the memory deficits in AD models of rodents^20,34^. After the bilateral IT injection into the MS/vDB or NBM, Tg and non-Tg mice (n = 100 for each mouse group) were administered intraperitoneally (i.p.) saline or low and high doses of Done and Riva (1 and 4 μmol/kg, respectively), and 30 min later they were subjected to the three-chamber test.

**Figure 3:**
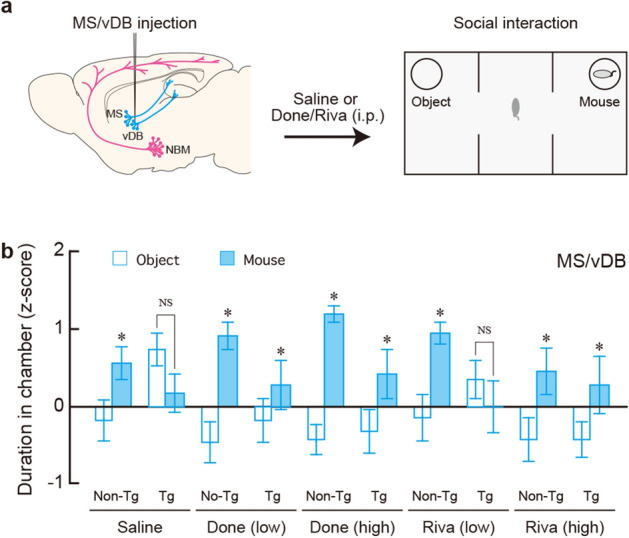
Drug effect on the deficit in social interaction in mice lacking MS/vDB cholinergic neurons. (**a**) Experimental design for a recovery experiment from the interaction deficit. Tg and non-Tg mice injected with IT solution into the MS/vDB were given systemic administration (i.p.) with saline, Done or Riva at low and high doses (1 and 4 μmol/kg, respectively), and 30 min later were used for the three-chamber test. (**b**) Mean z-score of the duration in the object and mouse chambers during the social interaction session. Data are presented as mean ± s.e.m. *n* = 10 for each group. *P < 0.05 vs the object chamber (two-way ANOVA with the repeated measure). NS, not significant.

The mice that received IT-injection into the MS/vDB were tested for the recovery of social interaction after the familiarization session (Fig. 3a). Following saline administration, the duration in the mouse chamber was significantly longer than that in the object chamber in the non-Tg mice, whereas the scores were similar between the two chambers in the Tg mice (Fig. 3b: group, *F*_1,18_ = 1.244, *P* = 0.279; chamber, *F*_1,18_ = 0.149, *P* = 0.704; interaction, *F*_1,18_ = 7.832, *P* = 0.502, two-way ANOVA; non-Tg, *F*_1,18_ = 5.072, *P* = 0.037; Tg, *F*_1,18_ = 2.909, *P* = 0.105, post hoc), confirming impaired social interaction in the Tg mice lacking the MS/vDB cholinergic neurons. After Done administration with either a low or high dose, the duration in the mouse chamber was significantly longer than that in the object chamber in both the non-Tg and Tg mice (Fig. 3b: group, *F*_1,18_ = 0.563, *P* = 0.463; chamber, *F*_1,18_ = 8.998, *P* = 0.008; interaction, *F*_1,18_ = 2.220, *P* = 0.154, two-way ANOVA for the low-dose; group, *F*_1,18_ = 2.980, *P* = 0.101; chamber, *F*_1,18_ = 20.46, *P* < 0.001; interaction, *F*_1,18_ = 2.624, *P* = 0.123, two-way ANOVA for the high-dose group). After the low-dose Riva administration, the duration was significantly longer for the mouse chamber compared to the object chamber in the non-Tg mice, but there was no significant difference between the two chambers in the Tg mice (Fig. 3b: group, *F*_1,18_ = 0.697, *P* = 0.339; chamber, *F*_1,18_ = 1.557, *P* = 0.229; interaction, *F*_1,18_ = 5.539, *P* = 0.030, two-way ANOVA; non-Tg; *F*_1,18_ = 6.476, *P* = 0.020; Tg, *F*_1,18_ = 0.614, *P* = 0.443, post hoc). Following the high-dose Riva administration, the duration was significantly longer in the mouse chamber compared to that in the object chamber in both kinds of mice (Fig. 3b: group, *F*_1,18_ = 0.113, *P* = 0.741; chamber, *F*_1,18_ = 4.512, *P* = 0.048; interaction, *F*_1,18_ = 0.064, *P* = 0.803, two-way ANOVA). These results indicate that the administration of ChEIs, except in the mice administered with a low dose of Riva, can restore the decline in social interaction generated by the dysfunction of MS/vDB cholinergic cell group.

**Figure 4:**
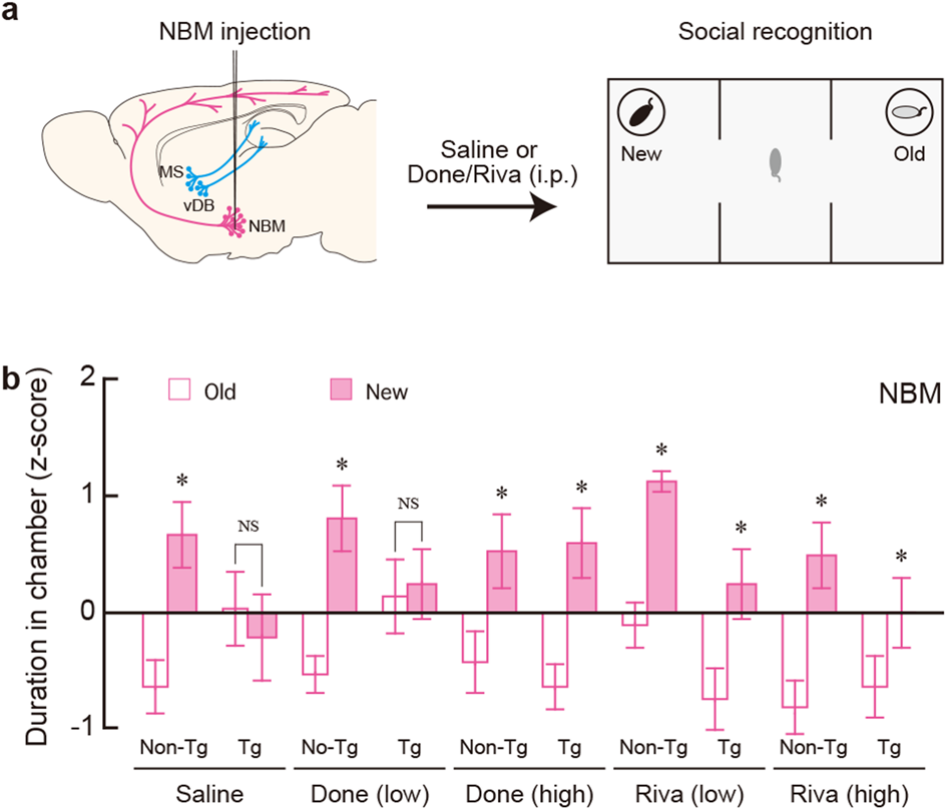
Drug influence on the impairment in social recognition memory in mice lacking NBM cholinergic neurons. (**a**) Experimental design for a recovery experiment from the memory impairment. Tg and non-Tg mice with IT injection into the NBM were administered i.p. with saline, Done or Riva with the low and high doses (1 and 4 μmol/kg, respectively), and then 30 min later subjected to the three-chamber test. (**b**) Mean z-score of the duration in the old and new mouse chambers during the social recognition memory session. Data are presented as mean ± s.e.m. *n* = 10 for each group. *P < 0.05 vs the old mouse chamber (two-way ANOVA with the repeated measure). NS, not significant.

The mice that received IT injection into the NBM were tested for the reversal of social recognition memory after the familiarization and social interaction sessions (Fig. 4a). Following saline administration, the duration in the new mouse chamber was significantly longer than that in the old mouse chamber in the non-Tg mice, while the values were similar between the two chambers in the Tg mice (Fig. 4b: group, *F*_1,18_ = 0.287, *P* = 0.599; chamber, *F*_1,18_ = 2.77.1, *P* = 0.161; interaction, *F*_1,18_ = 4.861, *P* = 0.041, two-way ANOVA; non-Tg, *F*_1,18_ = 6.723, *P* = 0.018; Tg, *F*_1,18_ = 0.276, *P* = 0.606, post hoc), ascertaining the impaired social recognition memory in the Tg mice deleting NBM cholinergic neurons. After the low-dose Done administration, the duration in the new mouse chamber was significantly longer than that in the old mouse chamber in the non-Tg mice, but there was no significant difference in duration between the two chambers in the Tg mice (Fig. 4b: group, *F*_1,18_ = 1.790, *P* = 0.198; chamber, *F*_1,18_ = 5.812, *P* = 0.027; interaction, *F*_1,18_ = 4.446, *P* = 0.049, two-way ANOVA; non-Tg, *F*_1,18_ = 10.212, *P* = 0.005; Tg, *F*_1,18_ = 0.046, *P* = 0.833, post hoc). After the high-dose Done administration, the duration was significantly longer for the new mouse chamber compared to the old mouse chamber in both the non-Tg and Tg mice (Fig. 4b: group, *F*_1,18_ = 0.128, *P* = 0.725; chamber, *F*_1,18_ = 11.865, *P* = 0.003; interaction, *F*_1,18_ = 0.174, *P* = 0.682; two-way ANOVA). Following Riva administration with the low/high doses, the duration in the new mouse chamber was significantly longer than that in the old chamber in both kinds of mice (Fig. 4b: group, *F*_1,18_ = 21.775, *P* < 0.001; chamber, *F*_1,18_ = 14.737, *P* = 0.001; interaction, *F*_1,18_ = 0.167, *P* = 0.201, two-way ANOVA for low dose; group, *F*_1,18_ = 0.752, *P* = 0.397; chamber, *F*_1,18_ = 8.845, *P* = 0.008; interaction, *F*_1,18_ = 0.948, *P* = 0.343, two-way ANOVA for high dose). These data indicate that, although the low dose of Done was ineffective, the administration of ChEIs can reverse disturbance in social recognition memory produced by NBM cholinergic dysfunction.

### Impact of cholinesterase inhibition on locomotor activity in mice lacking cholinergic cell groups

**Figure 5:**
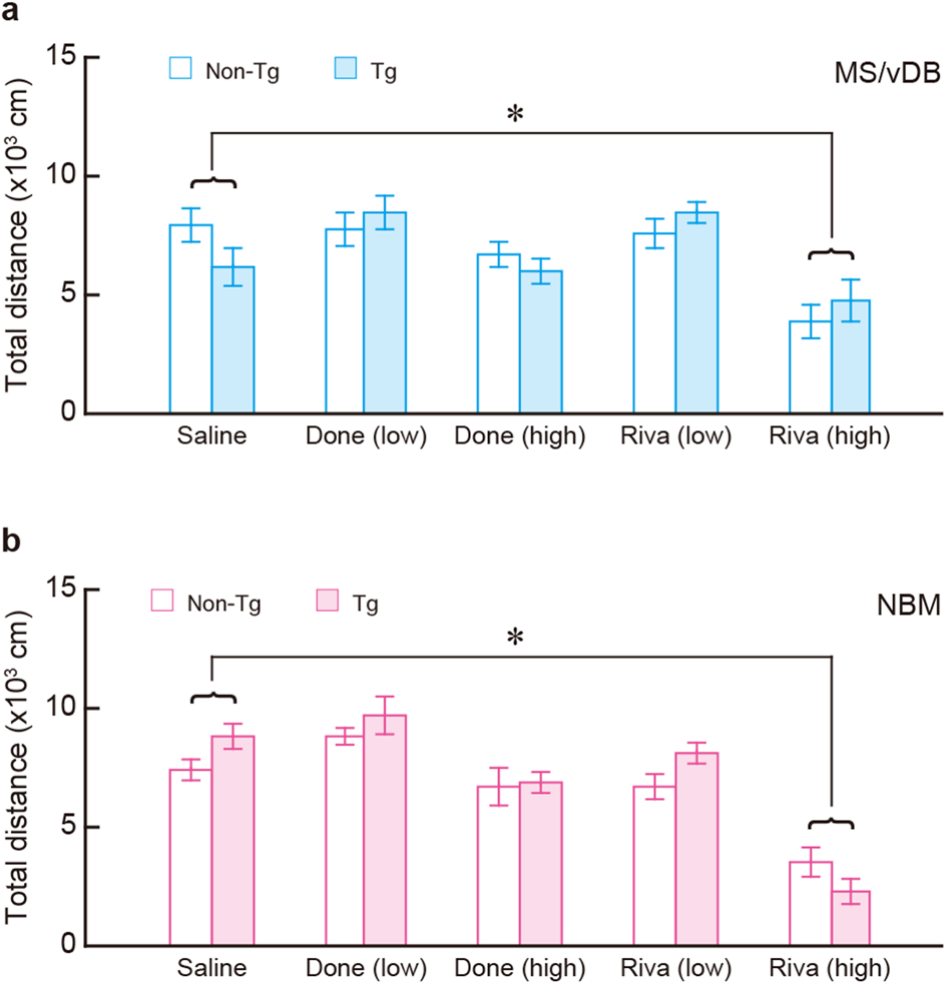
Locomotor activity in mice lacking basal forebrain cholinergic neurons after drug administration. Mice with IT injection into the MS/vDB or NBM were administered i.p. with saline, Done or Riva at low and high doses (1 and 4 μmol/kg, respectively), and 30 min later were moved to the familiarization session in the three-chamber test. (**a,b**) Mean travelled distance in the apparatus during the familiarization session in the injected mice into the MS/vDB (**a**) or NBM (**b**). Data are presented as mean ± s.e.m. *n* = 10 for each group. *P < 0.05 vs saline administration (*Bonferroni* method).

Additionally, we examined the effect of systemic ChEI administration (i.p.) on the locomotor activity of the Tg and non-Tg mice with IT injection into the MS/vDB or NBM. To evaluate the locomotor activity of the mouse groups, we measured the travelled distance during the familiarization session of the mice used for the recovery experiments mentioned above. For the mice that were given IT injection into the MS/vDB, the travelled distance was comparable among the mouse groups that were administered saline, low/high doses of Done, and low dose of Riva. The distance was significantly reduced only in the mice administered a high dose of Riva compared to saline administration, although it did not differ between the Tg and non-Tg mice with all conditions of the administration (Fig. 5a: group, *F*_1, 90_ = 0.008, *P* = 0.928; drug, *F*_4, 90_ = 10.924, *P* < 0.001; interaction, *F*_4, 90_ = 1.747, *P* = 0.147, two-way ANOVA, *Bonferroni* method, *P* < 0.05). For the mice that received IT injection into the NBM, similarly to the MS/vDB injection, the travelled distance was indistinguishable among the administration with saline, low/high doses of Done, and low dose of Riva. In addition, the distance was significantly decreased only in the administration with the high dose of Riva compared to saline administration, but there was no difference in the distance between the two kinds of mice with all administration conditions (Fig. 5b: group, *F*_1, 90_ = 2.879, *P* = 0.093; drug, *F*_4, 90_ = 36.167, *P* < 0.001; interaction, *F*_4, 90_ = 1.799, *P* = 0.136, two-way ANOVA, *Bonferroni* method, *P* < 0.05). These data show that locomotor activity in the apparatus during the familiarization session was decreased in the mice only when the high dose of Riva was administered, regardless of the presence or absence of cholinergic neurons in the MS/vDB or NBM, suggesting that high-dose administration of Riva may have a side effect of reducing locomotion in mice.

## Discussion

In the present study, we aimed to clarify the roles of basal forebrain cholinergic cell groups in social behaviour. We performed selective targeting of MS/vDB and NBM cholinergic cell groups in mice with IT-mediated cell targeting, and examined the social behaviour of these mice by using the three-chamber test. Selective elimination of MS/vDB cholinergic neurons resulted in no preference for the chamber with the social stimulus (mouse) over that with the non-social stimulus (object), indicating impairment in social interaction. The elimination of NBM cholinergic neurons displayed no preference to the chamber with the novel social stimulus (new mouse) against the familiar one (old mouse), implying the impaired social recognition memory. The deficit in social interaction or social recognition memory by dysfunction of different cholinergic cell groups was restored by systemic administration with appropriate doses of ChEIs including Done and Riva that activate the cholinergic systems. Our results reveal that MS/vDB and NBM cholinergic cell groups have critical and dissociable roles in social interaction and social recognition memory, respectively.

Our cell targeting technology with anti-Tac(Fv)-based recombinant IT enabled us to conduct the selective, efficient elimination of basal forebrain cholinergic neurons without any influence on the number of parvalbumin-positive GABAergic neurons and tissue damage around the injection sites and the elimination of these neurons did not affect the locomotor activity^20^. In contrast, other previous studies reported that 192 IgG-saporin and p75-conjugated saporin occasionally induce non-specific damage of GABAergic neurons and extensive tissue injury in the basal forebrain^35–38^, in addition to producing the limited lesion of neurons^39–41^. Some of these studies also reported that cholinergic ablation with saporin-conjugated neurotoxins leads to an increase in locomotion^41–44^. The inconsistency in locomotor activity after cholinergic ablation between previous studies and the current study suggests that non-specific damages in the basal forebrain regions affected locomotor activity in the previous studies.

In this study, although systemic administration of high doses of Riva recovered impaired social interaction or social recognition memory in mice lacking different cholinergic cell groups in the basal forebrain, this treatment generated a decrement in locomotor activity in both the Tg and non-Tg mice that received IT injection, suggesting the potential of high-dose Riva to reduce locomotion, irrespective of the presence or absence of cholinergic neurons. One possible explanation for this observation is that the systemic administration may influence the activity of cholinergic interneurons in the striatum, and that these interneurons may be more susceptible to high doses of Riva. Ablation of striatal cholinergic interneurons is known to cause hyperactivity in mice, suggesting an inhibitory role of these neurons in locomotion^45,46^. Therefore, striatal cholinergic activation may be involved in the hypoactivity observed after systemic administration of high-dose Riva. Another possibility may be related to the trait on behavioural effects of butyrylcholinesterase, which is inhibited selectively by Riva. Both Done and Riva inhibit acetylcholinesterase, and Riva possesses an additional inhibitory activity against butyrylcholinesterase^47^. Butyrylcholinesterase-positive neurons are found in the forebrain and brain stem, including the thalamus and tegmental areas^48,49^. The thalamus and tegmental areas constitute parts of the cortico-basal ganglia loop, which plays a central role in the control of locomotor activity^50,51^. The effect of high-dose Riva may influence, partially through the inhibition of butyrylcholinesterase, the activity of the cortico-basal ganglia loop, leading to reduced locomotor activity; however, the neural mechanism that explains butyrylcholinesterase inhibition affecting the cortico-basal ganglia circuit remains unknown.

Our findings from the three-chamber test show that MS/vDB cholinergic neurons are involved in social interaction but are not involved in social recognition memory. There are prominent anatomical connections between MS/vDB cholinergic neurons and the dorsal and ventral hippocampus^23^. Social interaction is reported to be increased by excitotoxic lesion of the whole hippocampal region^52^ and, especially, the ventral hippocampus in rats^53^. Lesion of the fimbria results in a reduction of social interdependency without affecting affiliative behaviours such as grooming, sniffing, and nosing^5^. These findings suggest that cholinergic activation in the septo-hippocampal pathway may mainly mediate the processing of social information during social interaction. In addition, several cholinergic neurons in the MS/vDB project to the medial prefrontal cortex^23^, and nicotinic acetylcholine receptor signaling in the medial prefrontal cortex appears to affect social contacts^54^. Therefore, social interaction may be also regulated by cholinergic activity in the medial prefrontal cortex. More detailed examination of the roles of cholinergic cell groups in various types of social interactions will provide clues for the elucidation of the neural mechanisms underlying social ability in mammals.

In addition, our previous study indicated that the lesion of the MS/vDB cholinergic cell group led to normal contact and habituation to objects, and that it disturbed spatial recognition memory but not object recognition memory^20^. This evidence excludes the possibility that the general attention and memory functions are impaired in mice lacking the MS/vDB cholinergic cell group. The impairment we observed in the social interaction of Tg mice may also be associated with dysfunction in processing social information rather than some changes in general attention and memory functions. However, social interaction is a broad and complex behavioural concept including memory and attention which take on social information, and it is inherently accompanied by memory (or habituation) and attention for other individuals. We need a more detailed analysis of the behavioural elements including memory and attention related to social information processing in the future.

In contrast to the MS/vDB, NBM cholinergic neurons were engaged in social recognition memory, but not in social interaction in the present study. Our findings support those of previous studies, which reported the damage of social recognition memory by systemic dysfunction of cholinergic system^56,57^. The cholinergic terminals of NBM distribute to the basolateral amygdala and the entire neocortical regions including the medial prefrontal cortex^24–26^. The basolateral amygdala and medial prefrontal cortex are reported to play an essential role in social recognition memory^58^. Such evidence suggests that cholinergic NBM projections may control the information processing during social recognition via the basolateral amygdala or medial prefrontal cortex. In addition to social recognition memory, NBM cholinergic neurons are also required for object recognition memory^20^. These requirements suggest that dysfunction of the NBM cholinergic system generally impairs recognition memory depending on various cues. It is unknown whether different types of cholinergic neurons in the NBM are involved in social and object recognition memory. Further investigation will be needed to clarify how this cholinergic cell group regulates processing of specific types of recognition memory through their target regions.

Ablation of cholinergic NBM neurons in rats by 192 IgG-saporin decreases the passive social contact, such as sitting or lying closely to each other, but increases active social contact as sniffing, grooming, and fighting^55^. This result shows the involvement of NBM cholinergic neurons in social contacts, which is not consistent with our results, which show the independency of NBM cholinergic lesions in social interaction. One reason that explains the inconsistency is because of the difference in the monitoring methods of social behaviour. Social interaction was evaluated by comparing relative duration in a chamber with a mouse with a chamber with an object in the current study, whereas it was monitored by examining multiple factors constituting social contacts in their study^55^. Alternatively, non-specific neuronal damages in the NBM after local injection of 192 IgG-saporin may have led to the complex changes in social contacts.

Early mnemonic impairments in AD are considered to mainly be caused by the dysfunction of cholinergic neurons^16–18^, but it is still uncertain whether the loss of cholinergic neurons in the basal forebrain is related to BPSD. Nonetheless, BPSD is an important clinical target for AD intervention^59^. In the present study, the elimination of cholinergic cell groups on MS/vDB and NBM in the basal forebrain differently resulted in the impairments of social behaviour which were restored by treatment with ChEIs. These data suggest that dysfunction of the cholinergic system in the basal forebrain may explain deficits in social ability associated with BPSD in AD. Our strategy provides a new Tg animal model for BPSD based on the selective loss of basal forebrain cholinergic neurons, not only for the dementia with recognition memory impairment^20^. This animal model will be useful for the elucidation of the neural mechanisms underlying cognitive impairments and for the development of approaches for diagnostic and therapeutic treatments.

## Methods

### Animals

*ChAT-IL-2Rα/mVenus* Tg mice that carried a gene cassette encoding IL-2Rα/mVenus followed by the SV40 early-gene polyadenylation signal downstream of the murine *ChAT* gene promoter were generated, and the Tg mice were identified using Southern blot hybridization or PCR with genomic DNA prepared from tail clips as described in our previous study^20^. The Tg and non-Tg littermates (n = 172 for each mouse group) were used for the following experiments. All animal experiments were approved and performed in accordance with the Guidelines for the Care and Use of Laboratory Animals established by the Animal Experiments Committee of Fukushima Medical University and Hiroshima University. This study was carried out in compliance with the ARRIVE guidelines.

### Intracranial injection

Surgery was conducted as described by Okada et al.^20^. Mice (8–10 weeks old) were anesthetized with sodium pentobarbital (50 mg/kg, i.p.) and subjected to bilateral intracranial injection of IT solution [20 μg/ml anti-Tac(Fv)-PE38 in PBS containing 0.1% mouse serum albumin]. For targeting of cholinergic neurons in the MS/vDB and NBM, IT solution or vehicle was randomly injected into 12 sites (0.2 µl/site) and six sites (0.3 µl/site) using the block randomization, respectively, through glass micropipette that was stereotaxically introduced by using the coordinates from an atlas of the mouse brain^60^. The anteroposterior, mediolateral and dorsoventral coordinates (mm) from bregma and dura were (1.1, ± 0.1, − 3.7), (1.1, ± 0.1, − 4.1), (0.8, ± 0.1, − 3.8), (0.8, ± 0.3, − 4.7), (0.6, ± 0.1, − 3.7), and (0.6, ± 0.1, − 4.2) for injection into the MS/vDB; and (− 0.4, ± 1.6, − 3.7), (− 0.7, ± 1.8, − 3.8), and (− 0.9, ± 2.0, − 3.8) for injection into the NBM (Supplementary Fig. S1). Injection was carried out at a constant flow rate of 0.1 µl/min with a microinfusion pump (ESP-36, EICOM), and the micropipette was left in situ for 2 min after each infusion.

### Drug treatment

Drug solution was prepared as described previously^20^. Donepezil hydrochloride (Sequoia Research Products Ltd.) and rivastigmine hydrogen tartrate (kindly provided by Novartis Pharma AG, Basel, Switzerland) were dissolved into saline at final concentrations of 0.1 and 0.4 mM. The mice randomly received the i.p. treatment of each drug solution (1 or 4 μmol/kg) or saline with the block randomization, 30 min before the behavioural testing.

### Histology

Immunohistochemistry and cell counts were performed as described^20^. Fixed brains were cut into sections (30-µm thick), and the sections were incubated with primary antibodies for ChAT (mouse, 1:1000, Millipore), and then with a biotinylated secondary antibody (anti-mouse IgG, 1:200, Jackson ImmunoResearch Laboratories). The immunoreactive signals were visualized by using a Vectastain Elite ABC kit (Vector Laboratories). For cell counts, the number of immune-positive cells in each area was counted in the representative four sections through the MS/VDB or NBM (the anteroposterior coordinates from bregma: 1.3, 0.9, 0.7, and 0.5 mm for the MS/VDB; and − 0.3, − 0.5, − 0.8, and − 1.0 mm for the NBM), and the total number of immunopositive cells was calculated.

### Behavioural analysis

The three-chamber test were conducted as described by Moy et al.^3^ with some modifications. Adult naïve male mice were group-housed in standard lab Plexiglas cages (225 × 338 × 140 mm, length × width × height, four mice per cage) on a 12-h light/12-h dark cycle and freely access to food and water. The experiments were conducted during the light period. After the surgery, the mice were given a 1-week recovery period. The experiment was conducted in a room with an illuminance level of 20 lx. The testing apparatus was made of clear Plexiglas and consisted of three open adjacent chambers, including a central chamber and two side chambers (each 200 mm length × 400 mm width × 225 mm height) separated by two clear plastic dividers and connected by open doorways (50 mm width × 85 mm height). Each animal’s behaviour was monitored using an overhead colour CCD camera (ALDP-292P, NADATEL Co., Ltd) mounted on the ceiling, approximately 740 mm above the field surface. The apparatus and cages were cleaned with 70% ethanol between animals.

The three-chamber test consisted of three 10-min sessions with 1-min intersession intervals. In the first session, for familiarization (Fig. 2a-i), the mice were allowed to habituate to the apparatus and freely explore it. Additionally, general locomotor activity was measured by the travelled distance in all chambers during this session. In the second session, for social interaction (Fig. 2a-ii), an unfamiliar C57BL/6 J male mouse (8–10 weeks old) was placed in a cylindrical steel-meshed cage (87 mm in height × 80 mm in diameter) in one of the side chambers (mouse chamber) and another identical empty cage (object chamber) was placed in the chamber on the other side. The positions of the mouse and object (empty cage) were counterbalanced between the subjects. The experimental mouse subjects freely explored freely all three chambers, and the measurements were the time durations they spent in each chamber. Preference for the mouse chamber over the object chamber was set to the index of social interaction. To alleviate the effect of the variation of baseline locomotor activity among individual animals, the data of the durations in each chamber were normalized into z-scores, according to the formula: $z=\frac{X-\bar{X}}{S}$, where $X$ is the raw score of duration in each chamber, $\bar{X}$ is the mean among the durations in all chambers, and $S$ is the standard deviation. In the third session, for social recognition memory (Fig. 2a-iii), the cage with the male mouse in the second session was kept in the same side chamber (old mouse chamber), and the empty cage was replaced by an identical cage with another unfamiliar C57BL/6 J male mouse (8–10 weeks old) (new mouse chamber). The experimental mouse subjects were again allowed to explore all chambers freely, and the duration in each chamber was measured. Preference for the new mouse chamber over the old mouse chamber was set to the index of social recognition memory. Individual data were also converted to z-scores, as described above. The travelled distance in the apparatus and duration in each chamber were automatically quantified by computer software (Smart 3; Panlab, S.L.U.).

After all behavioral tests, the mice were anesthetized with sodium pentobarbital and perfused transcardially with PBS, followed by 10% formalin. The brain sections were stained with cresyl violet for verification of the placement sites of the glass micropipette. Histological examination confirmed the placement sites within the target brain regions.

### Statistical analysis

To decide the sample size, we employed the similar numbers of animals to those that were reported in the previous publications^3,20^, which are generally accepted in the research field. In this study, the histological and behavioural data obtained from all animals used were provided for the statistical analysis. For statistical comparisons, ANOVA, followed by post hoc analysis and *Bonferroni* test, was used with statistical significance set at *P* < 0.05 (SPSS 22.0, IBM). Two-way ANOVA with repeated measure was used for the analysis of within-subjects design. All data were displayed as mean ± s.e.m.

## Supplementary Information

Supplementary Figures.

## Data Availability

The datasets generated and analyzed in the current study are deposited at Mendeley Data: https://data.mendeley.com/drafts/g2sm4hthvx.
