# Supplementary material for: Different cholinergic cell groups in the basal forebrain regulate social interaction and social recognition memory
Source: Sci Rep. 2021 Jun 30;11:13589. doi: 10.1038/s41598-021-93045-7 (PMC8245640; doi:10.1038/s41598-021-93045-7)
Supplement: Supplementary file 1 — Supplementary Figures. [file 41598_2021_93045_MOESM1_ESM.pdf]

SUPPLEMENTARY INFORMATION

Title: Different cholinergic cell groups in the basal forebrain regulate social interaction and social recognition memory

Authors: Kana Okada, Kayo Nishizawa, Tomoko Kobayashi, Shogo Sakata, Kouichi Hashimoto and Kazuto Kobayashi

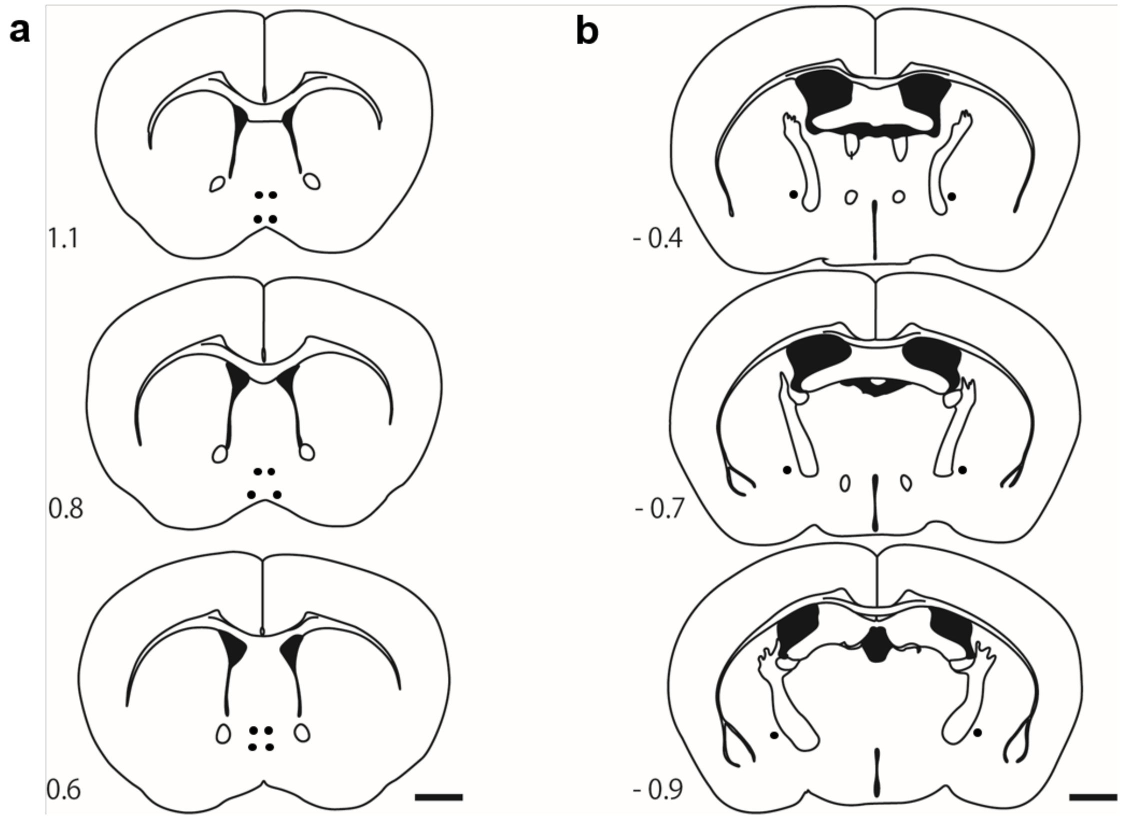

Supplementary Figure S1. Schematic illustration of the intracranial injection sites for stereotaxic surgery into the MS/vDB (**a**) or NBM (**b**). Black circles indicate coordinates for the injection. The anteroposterior coordinates (mm) from bregma are shown. Scale bars: 1 mm.

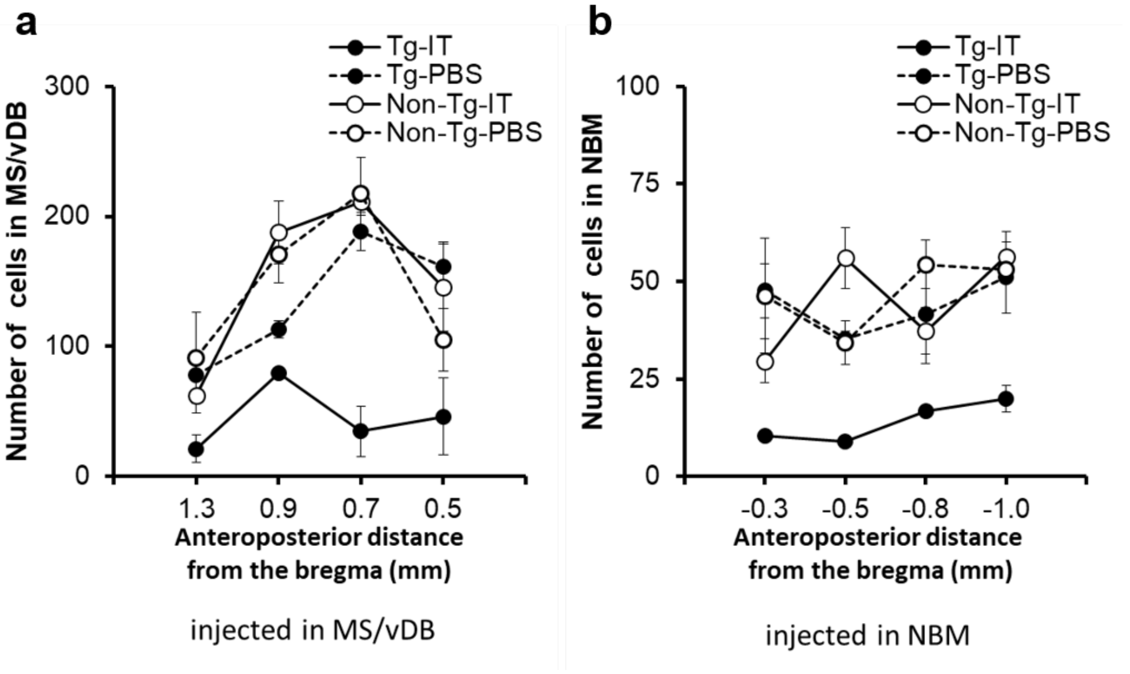

Supplementary Figure S2. Cell counts of ChAT-positive neurons along with the anteroposterior axis after intracranial injection. Number of ChAT-positive cells in the MS/vDB (distance from the bregma: 1.3, 0.9, 0.7, and 0.5 mm) in the MS/vDB-treated groups (**a**) and that in the NBM (distance from the bregma: −0.3, −0.5, −0.8, and −1.0 mm) in the NBM-treated groups (**b**). The cell number in the MS/vDB and NBM was significantly different among all 4 sections used for the immunostaining (repeated two-way ANOVA; group: $F(3, 8) = 26.741$, $P < 0.001$, section: $F(3, 24) = 9.220$, $P < 0.001$, interaction: $F(9, 24) = 1.528$, $P = 0.194$ for the MS/vDB; and group: $F(3, 8) = 11.316$, $P = 0.003$, section: $F(3, 24) = 1.753$, $P = 0.183$, interaction: $F(9, 24) = 1.103$, $P = 0.398$ for the NBM), showing a significant decrease in the cell number along the anteroposterior axis in the IT-injected Tg group as compared to each of other 3 groups in the MS/vDB and NBM (*Bonferroni* method, $P < 0.05$ for both regions). Data are presented as mean ± s.e.m.
